# Contributions of Medications, Physical and Hydrotherapy Programs in Reducing Endothelial Dysfunction in Hypertensive Patients

**DOI:** 10.3390/jfmk10020150

**Published:** 2025-04-28

**Authors:** Roxana Cristina Rad Bodan, Adina Octavia Dușe, Eniko Gabriela Papp, Răzvan Marian Melinte, Minodora Andor

**Affiliations:** 1 Doctoral School, Victor Babeş University of Medicine and Pharmacy, No. 2 Eftimie Murgu Square, 300041 Timișoara, Romania; roxana-cristina.rad@umft.ro; 2 Department of Balneofiziokinetotherapy and Medical Recovery, Faculty of Medicine, Dimitrie Cantemir University Târgu-Mureș, No. 3-5 Bodoni Sandor, 540545 Târgu-Mureș, Romania; eni_papp@hotmail.com; 3 Multidisciplinary Heart Research Center, Victor Babeş University of Medicine and Pharmacy, No. 2 Eftimie Murgu Square, 300041 Timişoara, Romania; 4 Department of Physical Medicine, Balneology and Rheumatology, Faculty of Medicine, Victor Babeș University of Medicine and Pharmacy, No. 2 Eftimie Murgu Square, 300041 Timișoara, Romania; duse.adina@umft.ro; 5 Department of Orthopedics and Traumatology, Iuliu Hațieganu University of Medicine and Pharmacy Cluj-Napoca, No. 8 Victor Babes, 400012 Cluj-Napoca, Romania; razvanmel@xnet.ro; 6 Department of Internal Medicine I, Victor Babeş University of Medicine and Pharmacy, No. 2 Eftimie Murgu Square, 300041 Timişoara, Romania

**Keywords:** hypertension, endothelial dysfunction, antihypertensives, physical therapy, hydrotherapy

## Abstract

**Background**: Hypertension is in first place in Europe among cardiovascular diseases. Worldwide, only 1 in 5 adults pursue proper treatment that controls their high blood pressure. Endothelial dysfunction is a marker that indicates the progression of hypertension. The study aims to evaluate the efficacy of antihypertensives and physical and hydrotherapy cardiovascular rehabilitation programs to control hypertension and improve endothelial dysfunction. **Methods**: A total of 100 patients with hypertension degree 1 (46 years ± 0.32) were divided into four homogenic groups. All subjects of the A, B, C and D groups benefited from recommendations for a healthy lifestyle; groups B, C and D also received antihypertensive medication; additionally, group C had a physical cardiovascular program and group D a cardiovascular hydrotherapy program. Several clinical endothelial damage markers and blood and urine parameters were registered, along with systolic and diastolic blood pressure, before and after 8 weeks of rehabilitation. **Results**: Group A registered a statistically significant decrease for 1 parameter LDL (*p* = 0.002). Group B showed statistically significant values for 14 parameters (*p* ≤ 0.05). The C and D groups registered significantly improved statistic values for 17 parameters each (*p* ≤ 0.0001). **Conclusions**: Groups B, C and D that were given antihypertensive medication during rehabilitation registered improved endothelial dysfunctional markers and controlled blood pressure values, compared with group A which was given only recommendations for a healthy lifestyle.

## 1. Introduction

The prevalence ranking of cardiovascular diseases worldwide places arterial high blood pressure (HBP) first, with 1.28 billion adults exhibiting the condition in 2023. Most importantly, the relationship of blood pressure values with the risks of cerebral, cardiovascular and renal accidents is emphasized [1]. The World Health Organization (WHO) deduced that almost 50% of hypertensives individuals are unaware of this health issue, and of the remaining 50%, most are ignoring the treatments; 1 in 5 adults pursue proper treatment to control their blood pressure values. The WHO target since 2010 has been to decrease the prevalence of HBP in 2030 by 33% [2].

Deeper research into hypertension markers proved their connections with the functioning of the vascular endothelium, whose role is to maintain the homeostasis of the vascular system. From there, more endothelium dysfunction indicates the possibility of cardiovascular disorder in most cases [3,4].

Interest in endothelial dysfunction research existed before the 1990s, and studies since then have contributed greatly to the understanding of cardiovascular pathologies, and implicitly of HBP [5,6,7]. After less than 10 years, the results of research on the clinical applicability of endothelial dysfunction markers for the prevention and detection of cardiovascular diseases appeared [8,9]. Current research brings endothelial dysfunction to the forefront for the creation of therapeutic programs for cardiovascular patients and even hypertensive patients [10,11,12,13,14].

Essential aspects of the studies in the 2020s show us the importance of physical activity, especially that based on aerobic exercises at a low intensity, to the result of preventing this risk of hypertension, as well as stabilizing and reducing blood pressure values [15,16,17]. In addition, though overlooked before, strength training has proved to be beneficial for cardiovascular patients [18].

The current literature repeatedly addresses various possibilities for the recovery of cardiovascular pathologies [19,20,21] because the number of patients in this situation has been continuously increasing in the last decade. High blood pressure occupies the first place among the risk factors for cardiovascular diseases. A different approach in testing and improving endothelial dysfunction correlated with high blood pressure is needed.

Endothelium dysfunction is a significant marker for the state of the blood vessels because primary atherosclerotic plaques are formed under the endothelium membrane, resulting in a narrowing of the lumen of the vessel; that may eventually damage or even rupture the membrane, which will lead to full occlusion [22,23].

Most of the interleukins are pro-inflammatory or anti-inflammatory, yet IL-6 is one of the cytokines that is both, along with IL-1, IL-2 and IL-12. Several studies proved the association of IL-6 with cardiovascular diseases [24], knowing that it is generated by endothelial cells, vascular smooth muscle cells, macrophages, monocytes and fibroblasts. IL-1, IL-2 and IL-12 are not used often in association with CVDs, at least not yet: IL-1 is a large family of cytokines, some of which have proved to have a connection with heart disease, but most with the myocardium due to their representation in epithelial and mesenchymal cells. Both IL-2 and IL-12 have been used mostly in the oncology domain [25,26,27,28].

Our findings, for the last decade, for physical therapy associated with endothelial dysfunction are poor, and in association with hydrotherapy are almost non-existent; most of them remain only in the physical exercise area [29,30,31].

The study seeks to find possibilities to improve endothelium function through different cardiovascular recovery methods for hypertensive patients, i.e., recommendations for a healthy lifestyle, antihypertensive medication and two diverse cardiovascular rehabilitation programs, one grounded in physical activity with land-based exercises and the other a hydrotherapy program.

## 2. Materials and Methods

### 2.1. Subjects

This prospective interventional study was conducted with 100 patients divided into four homogeneous groups, named A, B, C and D, with 25 individuals each.

From the records of cardiologists, a total of 275 patients with hypertension degree 1 were collected in order to obtain their agreement to analyze their personal medical files. Recruitment took place between January and February 2023 from Mureș county Romania; at their regular check-up, patients were asked to sign an informed consent form so that we were able to analyze their medical records.

The inclusion criteria were informed consent signed by the patients with agreement to active participation in the program if needed, diagnosis of hypertension degree 1 [32] and age of over 30 and up to 60 years old.

The exclusion criteria were other associated chronic diseases, dermatological or other skin pathology that may be contraindicated in physical or hydrotherapy, medication treatments that can influence BP values (contraceptives, diet pills, nasal decongestants, addiction, drugs, excess of tea, immunosuppressants, etc.), pregnancy, postpartum period, difference in BP values greater than 15 mmHg between arms, the patient not signing the informed consent form or the patient not agreeing to participate actively in the program sessions. There were nine homogeneous criteria: family history of hypertension, smoking, overweight, active lifestyle, active job, blood pressure values different between arms, gender, menstruating and menopause.

**Figure 1:**
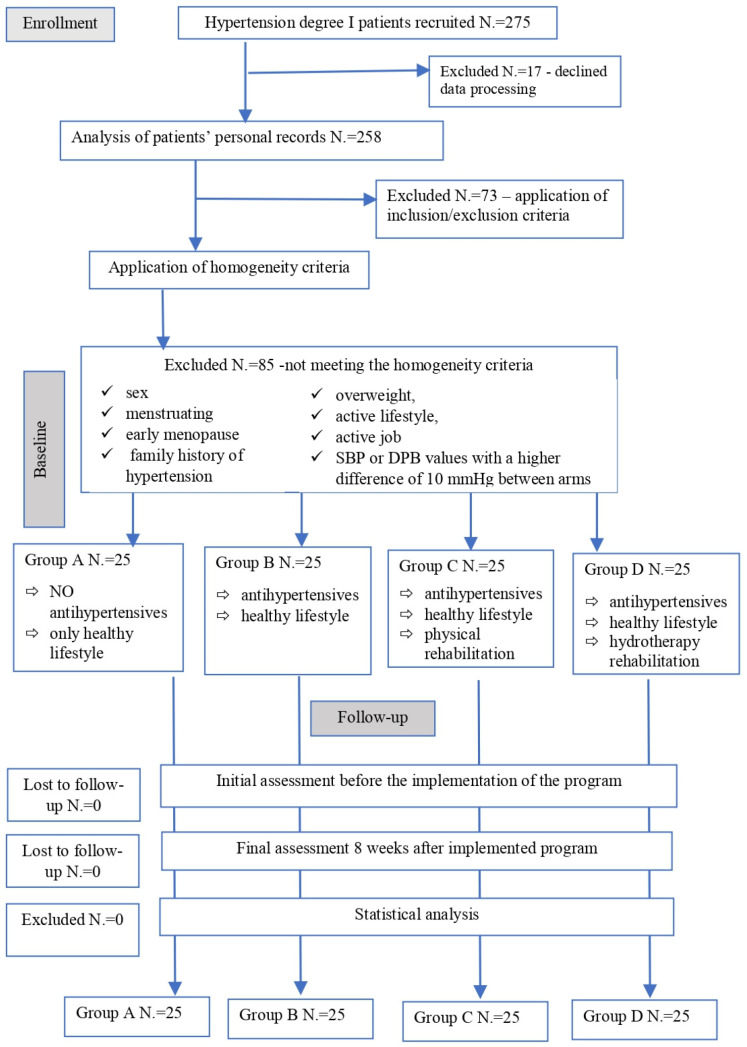
Flowchart.

After going through the inclusion/exclusion criteria, the nine homogenous factors based on cardiovascular risk were applied to ensure the homogeneity of the 4 groups. A total of 100 subjects remained in the program, and their results were taken into consideration for analysis. (Figure 1).

The recruited subjects were informed about all aspects regarding the study, i.e., the purpose, the procedures, the collection and possessing of data, the direct implication of the recovery program and the possibility to revoke their free consent at any time. All participants provided written informed consent.

### 2.2. Study Design

The study seeks to find possibilities to improve endothelium function through different recovery programs for hypertensive patients. Group A benefited exclusively from recommendations for a healthy lifestyle, while group B received antihypertensive medication in accordance with a cardiologist’s prescription along with the same recommendations for a healthy lifestyle presented in the study. Group C and group D, in addition to medication and recommendations for a healthy lifestyle, benefited from two different cardiovascular rehabilitation programs; group C underwent physical activity with land-based exercises, and for group D an hydrotherapy intervention.

### 2.3. Lifestyle Change

All four groups (A, B, C and D) benefited from information about the importance of changing their lifestyle. The most significant lifestyle change has been demonstrated to be losing weight, followed by reduction of salt ingestion, exercise and physical activities, diminishing alcohol consumption and quitting smoking. The eradication of noxious habits will prevent coronary, cerebrovascular and renal disorders for hypertensive patients. Above all, in accordance with the study’s purpose, choosing a healthier lifestyle may lead to controlled BP and even lowered values. We provided to all patients a questionnaire about their daily routine, and along with the information from their medical charts, we were able to give more specific information about what they need to improve in their lives. The questionnaires were given to the patients after signing the consent form, to be filled in by them at home; once completed, they brought them to the cardiologists’ offices or sent them by email. Based on their answers, along with the medical information we had, we were able to give them individual advice about what they should improve for their cardiovascular health, along with a printed plan with general criteria. This consultation was carried out together with the initial assessment of the patient. The printed plan included reducing salt consumption (low-sodium diet), restriction/moderation of alcohol consumption, a healthy diet combination based on vegetables and white meat, maintaining optimal weight or weight loss if needed, daily physical activity, smoking cessation, stress management and daily physical activity if possible or at least 3 times a week [33,34,35,36].

### 2.4. Antihypertensive Medication

The patients from groups B, C and D benefited from an individual antihypertensive medication plan according to their needs, prescribed by cardiologists. Based on the latest guidelines, a practical algorithm was developed for pharmaceutical treatment consisting of a combination of an angiotensin-converting enzyme inhibitor (ACEi) or angiotensin receptor blocker (ARB) with diuretics or low-dose non-dihydropyridine calcium channel blockers [37].

Patients included in group A did not receive prescribed medication during the 8-week program. This decision made by the cardiologists was based on the anamnestic data: with hypertension not higher than degree 1 and when there were no high-cardiovascular-risk factors that could threaten the patient’s life, drug treatment could be postponed temporarily [32].

Drug treatment of hypertension is based on very strong evidence, supported by the largest number of randomized outcome-based trials in clinical medicine. Meta-analyses of these studies conducted on hundreds of thousands of patients have shown that a 10 mmHg reduction in SBP or 5 mmHg reduction in DBP is associated with significant reduction in all major cardiovascular events by 20%, all-cause mortality by 10–15%, stroke by 35%, coronary events by 20% and heart failure by 40%. Prescribed antihypertensive medication by the cardiologist is intended to control blood pressure values and prevent organ damage, i.e., the occurrence of cardiovascular complications (cerebral, myocardial or renal). Optimal characteristics of medication treatment should encompass evidence linked to population characteristics, proof that it prevents morbidity and mortality, administration of one single pill which should control BP values for 24 h and is well-tolerated, affordability and ease of access for all patients [32].

### 2.5. Cardiovascular Physical Therapy

The 8-week program included 20 sessions of physical therapy for group C with a frequency of 2–3 times per week and took place in a specialized rehabilitation clinic: Rheum-Care Fondation Târgu Mureș, Romania. Three smaller groups were formed from group C in order to obtain the finest-organized sessions, enable better supervision and obtain the best outcome.

The maximum effort capacity was evaluated during the specialized cardiology consultation using electrocardiography exercise tolerance testing. According to the results, the interventional programs were carried out safely. Patients were instructed on the appearance of signs of extreme fatigue, which should not be reached, and when necessary the pulse and the blood pressure were monitored. The interplay between the number of repetitions, the duration of each exercise and the breaks in between were carried out in an adaptable and individualized manner for the patients to ensure the achievement of the moderate level of exertion required by the programs.

The goal was to create a pattern, constituted from the three sections, pursued during every session. The sections combined lasted for 50–80 min and were designed to reach 75% of each patient’s effort capacity, as follows:

Section 1 was constituted by warm-up exercises in order to prepare the body for effort: 10–15 min of stretching and mobilization of all body segments to activate the body’s circulatory system, without inducing tiredness; the aim of this section is to prevent incidents that may occur during the second part.
Section 2 is the main part, the exertion for 30 to 45 min in order to reach 75% of their effort capacity; to increase patients’ interest and to avoid boredom, a circuit was built on three main stations, i.e., walking on treadmill, ergo-bike and climbing stairs, as well as other types of physical exercise in between stations that stimulate all body parts, every muscle group and the entire cardiovascular system. This circuit was improved and modified every time it was necessary in order to fulfill all patients’ needs and to obtain the best rehabilitation feedback.
Section 3 involved recovery after effort with respiratory and relaxation methods in order to remove fatigue. This consisted of a minimum of 15 and up to 20 min of combined techniques between active relaxation and respiratory exercises: slow mobilization of the limbs and trunk in the rhythm of breathing, sometimes with isometric pauses that help release accumulated muscle tension; for the last 10 min, full body relaxation may be induced using different techniques like those of Schultz or Jacobson [38,39].

To sum up, each session included moderate intensity exercise, 75% of the maximum exertion previously established, for all body segments. The adaptability of the program through dosage allowed us to ensure at least a moderately increased metabolism; all muscle groups are included in each session, achieving local vasodilatation and a decrease in peripheral resistance; the pace is moderate with effective relaxation breaks to avoid going over the average threshold of physical demand [40,41].

### 2.6. Cardiovascular Hydrotherapy Program

Group D participated in a hydrotherapy program carried on at a specialized recovery center: Physiotherapy Balneo Center-Apollo Wellness Club Sângiorgiu de Mureș, Romania. The program consisted of 20 sessions distributed over a period of 8 weeks, with a frequency of 2 to 3 meetings per week.

One session had a duration of 50–80 min and was structured in three sections in order to reach 65% of each patient’s effort capacity, as follows:

Section 1 consisted of 15–20 min of preparing the patients for effort by using auxiliary means of physical therapy: magnetotherapy with lumbar and cervical applications; four cellular galvanic baths at a temperature of 37–38 °C with negative polarity in the lower limbs and positive in the upper limbs; bath in a pool with warm sodium chloride mineral water; light bath with dry air by infrared irradiation; dry air sauna; and infrared sauna.
Section 2 was the main part of the exercise: in a pool with regular water, using its resistance and specific floating devices, all body parts were stimulated along with the whole cardiovascular system. A circuit was built, consisting of different stations, i.e., walking, cycling and climbing stairs underwater, combined with other types of physical exercise for at least 30 and up to 40 min.
Section 3 involved lower intensity practice in order to induce recovery and relaxation after the effort, consisting of 15–20 min of freestyle swimming combined with floating; the finest way to achieve maximal relaxation was by using floating devices for all patients, even for those who proved to have very good swimming skills.

The effort was at a medium level, meaning 65% of the maximum effort capacity previously evaluated in the cardiologist’s office, because exercising in water allows gravitational discharge, with the advantage that patients do not feel the tiredness so quickly, even though all their muscles and joints are working. An accurate dosage of the session in accordance with the signs of fatigue and monitoring of pulse and blood pressure allowed us to reach and maintain the exertion exercises at 65% of maximum effort. Exercises in the water improve general and peripheral circulation, the return effort is greater, and the temperature of the water being up to 37 °C is definitely stimulating for the circulatory system and provides high-level relaxation [42].

### 2.7. Parameters Evaluated

Each and every patient included in the study partook in the same investigations and benefited from an initial assessment—before the implementation of the program—and a final assessment after 8 weeks of the predeterminate treatment program.

An assistant that did not know the study design was trained only to evaluate all the participants and to transfer all the data. During evaluation, the subjects were not allowed to disclose which program they followed. All the testing data and the samples were collected by the same assistant and transferred to the same clinic to be processed. Approved professional medical devices were used for data recording: blood pressure monitors P-200) and pulse oximeter (PM-60 Mindray) for oxygen saturation and heart rate respectively.

The study aims at evaluating the efficacy of maintenance of antihypertension medication associated with cardiovascular physical therapy and hydrotherapy in order to improve endothelial dysfunction and control blood pressure values.

Therefore, the primary objective of this study was to decrease blood pressure values, SBP and DBP, along with asymmetric dimethylarginine (ADMA), the inhibitor of neuronal nitric oxide synthase (nNOS), and the anti-endothelial cell antibodies (AECA) that affect endothelial cells.

In addition, the enhancement of biological markers was pursued:

- Improving the lipid profile by increasing protective lipid reactions of high-density lipoprotein cholesterol (HDL) and decreasing atherogenic fractions of low-density lipoprotein cholesterol (LDL);
- Improving the platelet level (PLT) to ensure aggregation function and decrease the risks of thrombosis;
- Stimulation of fibrinolysis, fibrinogen (FI) and thrombus destruction;
- Lowering the inflammation markers from blood and urine, interleukin-6 (IL-6), leukocyte (WBC), neutrophils (NEU) and C-reactive protein (CRP);
- Controlling early markers of renal disorders caused by hypertension: serum creatinine (sCr), microalbuminuria (MA) and urinary creatinine (uCr), as well as the urinary albumin/creatinine ratio (uACR).

### 2.8. Statistical Analysis

The GraphPad Prism V.9.0 software was used for statistical processing, applying the Wilcoxon test for comparison of non-parametric data (results being expressed as median—interquartile range) and Student’s *t*-test for comparison of parametric data (results being expressed as mean ± standard deviation) within each group. For the comparison of all four study groups, ANOVA and the Kruskal–Wallis tests were used, based on the type of data (parametric or non-parametric). For post-hoc evaluation, we used Tukey and Dunn’s multiple comparison test. The chi-square test and the Fisher test were used to compare proportions for the AECA parameter. The statistically significant results were those with *p* ≤ 0.05.

## 3. Results

### 3.1. Initial and Final Intergroup Analysis

**Figure 2:**
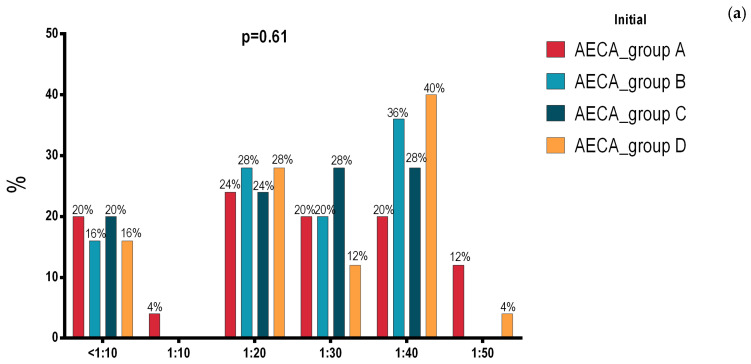

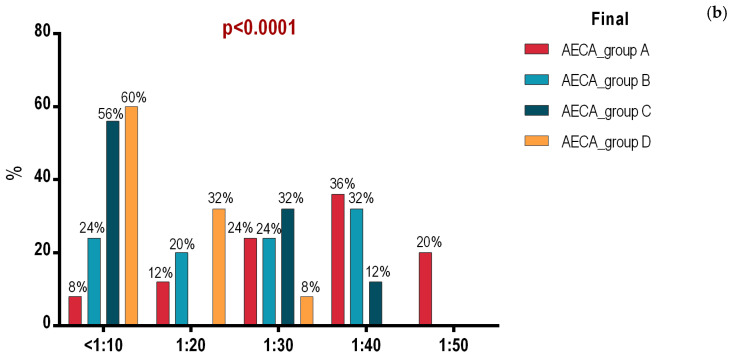
(**a**) Initial assessment of AECA for all four groups A, B, C and D; (**b**) Final assessment of AECA for all four groups A, B, C and D; AECA: anti-endothelial cell antibodies; *p* ≤ 0.05 was considered significant.

Following the comparison among all four groups A, B C and D, the baseline at the initial assessment, analyzed using the statistical ANOVA test, showed no significant statistical difference for 11 parameters, *p* > 0.05 (Table 1). Figure 2a shows that the initial assessment, analyzed via ANOVA, for anti-endothelial cell antibodies (AECA) had no statistically significant intergroup differences, *p* = 0.61.

However, the same analysis revealed significant differences for the following five variables: HGB, WBC, NEU, FI and uACR, with *p* < 0.05. Pairwise comparison showed a statistically significant differences for WBC between groups A and B (*p* = 0.005); for NEU among groups A and B and also among groups A and D (*p* = 0.02); for FI between groups A-C (*p* = 0.01); for HGB among groups A and C and also among groups A and D (*p* = 0.0003); and for uACR between groups A and D as well as between B and D (*p* = 0.01).

Figure 2b presents the final testing, following 8 weeks of the implemented programs and assessment, for anti-endothelial cell antibodies (AECA), with statistically significant differences among groups, *p* < 0.0001. Further analysis, realized with chi-square and Fisher’s tests for comparing proportions, highlights the best outcome for group D, which achieved 60% for the value < 1:10; group C is close, with 56% of the same value; group B registered a small increase of the percentage from 16% to 24%, and group A indicated a decrease from 20% to 8% (Figure 2b).

After 8 weeks, at the final assessment, the comparison of the four groups using the ANOVA analysis highlights significant statistical differences for seven variables (SBP, DBP, WBC, NEU, HDL, LDL and ADMA) when comparing groups C and D to groups A and B (*p* values < 0.05). Pairwise comparison showed statistically significant differences for several parameters among different groups (Table 2).

The hydrotherapy program combined with recommendations for a healthy lifestyle and antihypertensive medication of group D and the physical therapy combined with recommendations for a healthy lifestyle and antihypertensive medication of group C both showed a statistically significant decreases when compared to group A, which benefited only from recommendations for a healthy lifestyle, for three variables: SBP, with *p* = 0.005; DBP, HDL, WBC and NEU, each with *p* < 0.0001; and LDL, with *p* = 0.0003.

Further, the interventional programs of groups C and D revealed statistically significant differences in the final assessment compared to group B, which received antihypertensive medication along with recommendations for a healthy lifestyle, for two parameters: DBP and HDL, both with *p* < 0.0001.

In addition, regarding asymmetric dimethylarginine (ADMA), group D showed a significantly lower median in comparison with group A (*p* = 0.01); regarding leukocytes (WBC), group C showed a significantly higher mean when compared with group B, *p* = 0.0001.

A notable decrease was registered in interleukin-6 for group D when compared with the other three groups A, B and C, but it was not statistically significant, *p* = 0.08. For the other eight parameters, the data showed no statistical significance among groups after 8 weeks of the program, *p* > 0.05.

### 3.2. Intragroup Analysis of Initial and Final Assessment in Groups A, B, C and D

Intragroup comparison of the initial and final testing revealed that the patients of group A had statistically significant differences for only 3 parameters from the 16 presented in Table 2, while group B registered significant differences for 14. The outcomes of groups C and D are to be taken into consideration, given the fact that they showed statistically significant differences for all of their 16 parameters

Group A registered a statistically significant increase after 8 weeks, with only recommendations for a healthy lifestyle, in both systolic and diastolic blood pressure, indicating the progression of hypertension: SBP with *p* = 0.01 and DBP with *p* = 0.0007. One parameter showed a statistically significant decrease at the final moment with *p* = 0.002: LDL (Table 3).

The comparison between data obtained from the initial and final measurements for group B, which was given antihypertensive medication along with lifestyle recommendations, shows statistically significant values for 14 parameters: AECA, SPB, HDL, LDL, CPR and NEU registered the same value (*p* < 0.0001), and DBP, ADMA, IL-6, WBC, PLT, HGB, sCr and uACR had significant *p* values between 0.0001 and 0.05 (Table 4, Figure 2b).

Even if the analysis result is at threshold of statistical significance (*p* = 0.05), the increase from initial to final testing for urinary creatinine (uCr) is notable, and it may represent the prevalence in patients of vascular complications in the kidneys.

The results of the comparison between the initial and final measurements of group C show significant differences between all the studied variables, with *p* < 0.0001 for 16 parameters (SBP, DBP, HDL, LDL, IL-6, CPR, WBC, NEU, PLT, FI, HGB, sCr, MA, uCr, Uacr and AECA) and *p* = 0.001 for ADMA (Table 5, Figure 2b).

A similar outcome to those of the previous groups was recorded for group D, with significant differences in all 17 studied variables from the initial moment to the final moment: *p* < 0.0001 for 16 parameters (SBP, DBP, ADMA, HDL, LDL, IL-6, CPR, WBC, NEU, FI, HGB, sCr, MA, uCr, Uacr and AECA), and *p* = 0.001 for PLT (Table 6, Figure 2b).

Both interventional groups—group C with the physical therapy exercise program and group D with the hydrotherapy rehabilitation program—registered statistically significant values for each of the 17 parameters. The blood pressure values, DBP and SBP, along with the AECA parameter decreased significantly; the HDL, WBC and NEU values increased, and the LDL, HGB, PLT, CRP, FI, sCr, MA, uCr, uACR, IL-6 and ADMA values decreased (Table 5 and Table 6, Figure 2b).

The significant increase in neutrophils in both groups C and D is the result of the exercise programs, i.e., physical activity and hydrotherapy. The values registered, although higher after 8 weeks of the activity programs, are still in the normal range; most certainly these are generally short-term spikes.

## 4. Discussion

The most considerable results were definitely, as previously described, those of groups C and D, which registered statistically significant values with *p* < 0.05 for seven parameters (SBP, DBP, ADMA, HDL, LDL, WBC and NEU) compared with groups A and B at the final evaluation. At this point, from those seven parameters named above, by comparing them between groups C and D, we observe that five of them were slightly lower for group D (SBP, DBP, ADMA, LDL, WBC, NEU), while only one was lower for group C (HDL), meaning that group D which benefited from the hydrotherapy rehabilitation program had the best outcome.

Very close to a statistically significant value was the IL-6 parameter, registering *p* = 0.08 in the final-evaluation ANOVA analysis between the four groups. For this parameter, group D registered once more the lowest value, compared with the other groups’ medians. Improving the early markers, such as IL-6, has an impact in reducing cardiovascular risks; furthermore, the implementation of recovery programs for the hypertensive population may prevent complications and the appearance of other pathologies (such as cerebral injuries, coronary accidents or diabetes).

Despite several studies on the lowering of endothelial dysfunction [43,44], the literature remains sparse in revealing differences in outcome between pharmaceutical and pharmaceutical-plus-physical-therapy groups, and even more scattered regarding hydrotherapy programs. Evidence remains weak, so we need to continue in order to see the best results for each category of patient.

The physical therapy group, group C, was second in performance for most of the parameters, following the hydrotherapy-using group D, but was still very close in the median and mean data. We hypothesized that the early detection of signs of endothelial damage can prevent the occurrence of cardiovascular complications and the progressing of hypertension. The results show that recovery programs, especially those combined with medication and physical activity, will control and stabilize the cardiovascular parameters in order to slow the progression of the diseases and to postpone as much as possible cardiovascular complications.

Group B, with no other interventions besides recommendations for a healthy lifestyle and antihypertensive medication, showed improvement compared with group A, but it was still not statistically significant according to ANOVA analysis, as in the case of interventional groups C and D. The two rehabilitation programs based on antihypertensive medication and physical activity showed that controlling the cardiovascular parameters is possible, even to the point of lowering most of them. A continuation of these programs will definitely slow the progression of disease; even if cardiovascular complications are imminent, as research has shown, they may occur much later than predicted and may be not as destructive and massive as they would have been.

Lack of medication and a physical rehabilitation program led to an increase of the SBP and DBP values for group A. Most likely the recommendations for a healthy lifestyle may not be enough to stabilize blood pressure values and other significant cardiovascular parameters.

In the first decade of the 2000s, studies showed that if cardiovascular patients exercise, the death rate may drop by 20% because physical activity improves their capacity for effort by lowering their BMI, which also brings down cholesterolemia and leads to controlled BP values [45].

We should acknowledge the importance of physical activity and admit that movement is nourishment for the mind, body and soul. Once we admit that, we can also see the connection between lowering the level of catecholamines released by the body under stress and reducing the sensitivity of the cardiovascular system to them.

The number of sessions is determined in accordance with the minutes of each session and its intensity and complexity. Sessions of 30 min should be performed 5–7 days/week, or programs with 75 min per week of intense activity should be combined with low–moderate intensity activity 2–3 times weekly [46]. All in all, to reduce blood pressure and cardiovascular risks, it is important to achieve the required number of minutes of physical activity within one week. The duration of physical training must also be considered, as several studies have indicated that improvements in patients’ health can be seen after eight weeks [47,48,49].

This underlines the acute need for an individualized and more complex treatment approach that leads us to design therapeutic programs that may be continued by patients with minimal specialist supervision. A supreme accomplishment would be to transform the physical program into a lifestyle routine; that would be a marker of global progress, a true step towards a healthier society. Recognizing the importance of exercise for a healthy cardiovascular system is definitely the step forward that humanity needs.

A limitation of the study is the period of the implemented programs. Even though the results show improvement on several levels, it remains to be seen if rehabilitation programs may reverse the endothelium damage in order to lower the BP values in order to change the classification of the patient from hypertension degree 1 to prehypertension.

The long-term objective, that the programs could decrease the number of hypertensive patients with cardiovascular complications, is not yet reached; an extension of the study is needed in order to determine if further improvements may occur, noting the importance of when and what kind of rehabilitation programs and lifestyle changes patients should choose.

Our recommendation is that further studies should have a higher number of patients in order to confirm these results. The focus should be not only on the risk factors, but also on how we can change them to protective factors which may in turn reduce the complications, i.e., sedentarism being replaced with physical activity (2–3 days/week, for approximately 150–240 min/week), processed food being substituted with healthier variants and consumption of alcohol being reduced to a minimum.

## 5. Conclusions

The 8-week cardiovascular rehabilitation program shows improved endothelial dysfunctional markers and significantly stabilized blood pressure values for all three groups, B, C and D, that had antihypertensive medication in their therapeutic scheme, compared with group A that had only recommendations for a healthy lifestyle. Prescribed medication must be prioritized in all recovery programs for endothelial dysfunction and hypertension.

Our study’s findings point to the cardiovascular hydrotherapy program pursued by group D as having the best results. This rehabilitation program, based on hydrotherapy procedures, is a new and promising method for endothelial dysfunction recovery for hypertensive patients. Additionally, these results demonstrate that hydrotherapy, previously avoided in hypertension, may have an efficacious impact on endothelial dysfunction markers and can decrease and stabilize blood pressure values. The study’s findings place group C, which followed a physical rehabilitation program, second with close outcomes to those of group D, underlining that an active rehabilitation program and early intervention leads to greater results. Hydrotherapy elements, which registered the best outcome, should be taken into consideration for all further personalized rehabilitation programs for endothelial dysfunction recovery in hypertensive patients.

## Figures and Tables

**Table 1:** Initial-evaluation ANOVA analysis of between the four groups.

	Group A	Group B	Group C	Group D	*p* Value	Sig. dif.
SBP	147.2 ± 8.76	150 ± 5.90	150.9 ± 6.25	150.5 ± 6.98	0.24	
DBP	94.12 ± 2.97	94.8 ± 2.21	94.4 ± 2.48	94.24 ± 2.5	0.79	
ADMA	123.0 (97–140)	115 (91.0–142.0)	134 (103.5–143.5)	123 (99–139)	0.76	
HDL	53.56 ± 11.80	48.96 ± 16.43	52.64 ± 13.12	56.96 ± 12.74	0.19	
LDL	111.4 ± 20.18	101.4 ± 28.85	107.3 ± 23.73	110.2 ± 18.80	0.41	
IL-6	2.35 (1.89–3.38)	2.47 (1.73–4.72)	3.16 (1.84–4.3)	2.68 (1.83–4.56)	0.29	
CRP	0.25 (0.16–0.41)	0.31 (0.22–0.47)	0.36 (0.22–0.46)	0.34 (0.17–0.44)	0.2	
WBC	5780 ± 1088	7481 ± 2127	6832 ± 8025	6325 ± 2028	0.005	A-B
NEU	3170 (2505–3530)	5151 (2230–6230)	4250 (3525–5455)	4770 (3870–5485)	0.02	A-B, A-D
PLT	236,000 (193,000–258,500)	226,000 (173,500–366,000)	285,000 (233,500–3,515,000)	280,000 (210,500–340,500)	0.11	
FI	2.56 (2.23–2.86)	3.1 (2.65–3.7)	3.5 (2.25–3.75)	3.3 (2.55–4.0)	0.01	A-C
HGB	13.15 ± 1.74	14.43 ± 1.97	15.2 ± 1.54	15.07 ± 1.34	0.0003	A-C, A-D
sCr	0.806 ± 0.26	0.84 ± 0.25	0.84 ± 0.17	0.84 ± 0.12	0.21	
MA	5.38 ± 2.59	5.42 ± 2.93	5.05 ± 2.43	5.2 ± 2.41	0.95	
uCr	192.3 ± 75.97	251.8 ± 87.72	221.5 ± 72.08	220 ± 63.78	0.056	
uACR	2.7 (1.5–3.3)	2.7 (1.85–4.65)	3.0 (2.25–4.5)	3.6 (2.3–5.9)	0.01	A-D, B-D

SBP: systolic blood pressure; DBP: diastolic blood pressure; ADMA: asymmetric dimethylarginine; HDL: high-density lipoprotein cholesterol; LDL: low-density lipoprotein cholesterol; IL-6: interleukin-6; CRP: C-reactive protein; WBC: leukocytes; NEU: neutrophils; PLT: platelets; FI: fibrinogen; HGB: hemoglobin; sCr: serum creatinine; MA: microalbuminuria; uCr: urinary creatinine; uACR: urinary albumin/creatinine ratio; Sig. dif.: significant differences after pairwise comparison, post hoc evaluation Tukey test for median—interquartile range and Dunn’s multiple comparison test for mean ± standard deviation; *p* ≤ 0.05 was considered significant.

**Table 2:** Final-evaluation ANOVA analysis between the four groups.

	Group A	Group B	Group C	Group D	*p* Value	Sig. dif.
SBP	150.1 ± 10.24	148.2 ± 5.95	144.8 ± 4.77	144.0 ± 4.43	0.005	A-C, A-D
DBP	95.12 ± 2.63	94.2 ± 2.36	91.92 ± 2.97	90.16 ± 2.52	<0.0001	A-C, A-D, B-C, B-D
ADMA	131.0 (98.5–143.0)	110 (90.50–140.0)	110.0 (92–121.0)	109 (85–114)	0.01	A-D
HDL	53.32 ± 11.73	51.16 ± 15.94)	68.52 ± 7.43	70.96 ± 8.33	<0.0001	A-C, A-D, B-C, B-D
LDL	108.7 ± 18.88	99.96 ± 28.53	88.2 ± 14.40	87.76 ± 10.18	0.0003	A-C, A-D
IL-6	2.15 (1.74–3.38)	2.35 (1.87–3.89)	2.14 (1.37–3.19)	1.6 (1.15–3.40)	0.08	
CRP	0.26 (0.19–0.26)	0.30 (0.20–0.46)	0.21 (0.11–0.84)	0.2 (0.10–0.30)	0.3	
WBC	5910 (5120–6775)	6830 (5715–9815)	8710 (7995–8710)	7890 (6845–9670)	<0.0001	A-C, A-D, B-C
NEU	3200 (2500–3870)	5140 (1970–6215)	5820 (5000–6665)	5690 (5230–7230)	<0.0001	A-C, A-D
PLT	235,000 (191,000–259,000)	223,000 (172,000–366,500)	255,000 (184,500–2,965,000)	255,000 (198,000–297,000)	0.68	
FI	2.59 ± 0.56	3.14 ± 0.94	2.70 ± 0.56	2.97 ± 0.59	0.11	
HGB	13.19 ± 1.96	14.3 ± 1.98	13.86 ± 1.04	13.66 ± 0.84	0.09	
sCr	0.79 ± 0.24	0.82 ± 0.26	0.74 ± 0.16	0.74 ± 0.13	0.44	
MA	5.37 ± 2.54	5.34 ± 2.95	4.52 ± 2.19	4.25 ± 1.99	0.37	
uCr	199.4 ± 76.04	235.1 ± 80.85	201.1 ± 67.78	204.2 ± 62.76	0.31	
uACR	2.5 (1.65–3.50)	2.6 (1.7–4.55)	2.60 (180–3.80)	2.50 (1.55–3.95)	0.89	

SBP: systolic blood pressure; DBP: diastolic blood pressure; ADMA: asymmetric dimethylarginine; HDL: high-density lipoprotein cholesterol; LDL: low-density lipoprotein cholesterol; IL-6: interleukin-6; CRP: C-reactive protein; WBC: leukocytes; NEU: neutrophils; PLT: platelets; FI: Fibrinogen; HGB: hemoglobin; sCr: serum creatinine; MA: microalbuminuria; uCr: urinary creatinine; uACR: urinary albumin/creatinine ratio; Sig. dif.: significant differences after pairwise comparison, post hoc evaluation Tukey test for median—interquartile range and Dunn’s multiple comparison test for mean ± standard deviation; *p* ≤ 0.05 was considered significant.

**Table 3:** Initial and final testing, intragroup comparison of group A.

	Initial	Final	*p* Value
SBP	147.2 ± 8.76	150.1 ± 10.24	0.01
DBP	94.12 ± 2.97	95.12 ± 2.63	0.0007
ADMA	118.6 ± 26.56	124.3 ± 30.70	0.14
HDL	53.56 ± 11.80	53.32 ± 11.73	0.16
LDL	111.4 ± 20.18	108.7 ± 18.88	0.002
IL-6	2.35 (1.89–3.38)	2.15 (1.74–3.38)	0.32
CRP	0.25 (0.16–0.41)	0.26 (0.19–0.26)	0.25
WBC	5780 (4870–6540)	5910 (5120–6775)	0.97
NEU	3170 (2505–3530)	3200 (2500–3870)	0.56
PLT	237,600 ± 63,984	235,560 ± 64,429	0.52
FI	2.60 ± 0.47	2.59 ± 0.56	0.79
HGB	13.15 ± 1.74	13.19 ± 1.96	0.88
sCr	0.80 ± 0.26	0.79 ± 0.24	0.34
MA	5.381 ± 2.59	5.37 ± 2.54	0.95
uCr	192.3 ± 75.97	199.4 ± 76.04	0.28
uACR	2.7 (1.5–3.3)	2.5 (1.65–3.50)	0.34

SBP: systolic blood pressure; DBP: diastolic blood pressure; ADMA: asymmetric dimethylarginine; HDL: high-density lipoprotein cholesterol; LDL: low-density lipoprotein cholesterol; IL-6: interleukin-6; CRP: C-reactive protein; WBC: leukocytes; NEU: neutrophils; PLT: platelets; FI: fibrinogen; HGB: hemoglobin; sCr: serum creatinine; MA: microalbuminuria; uCr: urinary creatinine; uACR: urinary albumin/creatinine ratio; *p* ≤ 0.05 was considered significant.

**Table 4:** Initial and final testing, intragroup comparison of group B.

	Initial	Final	*p* Value
SBP	150 ± 5.90	148.2 ± 5.95	<0.0001
DBP	94.8 ± 2.21	94.2 ± 2.36	0.002
ADMA	115 (91–142)	110 (90.50–140.0)	0.006
HDL	48.96 ± 16.43	51.16 ± 15.94)	<0.0001
LDL	101.4 ± 28.85	99.96 ± 28.53	< 0.0001
IL-6	2.47 (1.73–4.72)	2.35 (1.87–3.89)	0.04
CRP	0.31 (0.22–0.47)	0.30 (0.20–0.46)	<0.0001
WBC	6850 (5765–9835)	6830 (5715–9815)	0.02
NEU	5151 (2230–6230)	5140 (1970–6215)	<0.0001
PLT	226,000 (173,500–366,000)	223,000 (172,000–366,500)	0.0005
FI	3.11 ± 0.74	3.14 ± 0.94	0.77
HGB	14.43 ± 1.97	14.3 ± 1.98	0.0002
sCr	0.84 ± 0.25	0.82 ± 0.26	0.04
MA	5.42 ± 2.93	5.34 ± 2.95	0.11
uCr	251.8 ± 87.72	235.1 ± 80.85	0.05
uACR	2.7 (1.85–4.65)	2.6 (1.70–4.55)	0.0001

SBP: systolic blood pressure; DBP: diastolic blood pressure; ADMA: asymmetric dimethylarginine; HDL: high-density lipoprotein cholesterol; LDL: low-density lipoprotein cholesterol; IL-6: interleukin-6; CRP: C-reactive protein; WBC: leukocytes; NEU: neutrophils; PLT: platelets; FI: fibrinogen; HGB: hemoglobin; sCr: serum creatinine; MA: microalbuminuria; uCr: urinary creatinine; uACR: urinary albumin/creatinine ratio; *p* ≤ 0.05 was considered significant.

**Table 5:** Initial and final testing, intragroup comparison of group C.

	Initial	Final	*p* Value
SBP	150.9 ± 6.25	144.8 ± 4.77	<0.0001
DBP	95 (92–96.5)	92 (90–95)	<0.0001
ADMA	125.8 ± 25.05	104.9 ± 19.84	0.001
HDL	52.64 ± 13.12	68.52 ± 7.43	<0.0001
LDL	107.3 ± 23.73	88.2 ± 14.40	<0.0001
IL-6	3.19 ± 1.56	2.415 ± 1.19	<0.0001
CRP	0.36 (0.22–0.46)	0.21 (0.11–0.84)	<0.0001
WBC	6820 ± 8025	8710 ± 10250	<0.0001
NEU	4250 ± 5455	5820 ± 6665	<0.0001
PLT	289,320 ± 70,871	251,760 ± 76,739	<0.0001
FI	3.5 (2.25–3.75)	2.8 (2.1–3.2)	<0.0001
HGB	15.2 ± 1.54	13.86 ± 1.04	<0.0001
sCr	0.84 ± 0.17	0.74 ± 0.16	<0.0001
MA	5.05 ± 2.43	4.52 ± 2.19	<0.0001
uCr	221.5 ± 72.08	201.1 ± 67.78	<0.0001
uACR	3.56 ± 1.94	2.88 ± 1.56	<0.0001

SBP: systolic blood pressure; DBP: diastolic blood pressure; ADMA: asymmetric dimethylarginine; HDL: high-density lipoprotein cholesterol; LDL: low-density lipoprotein cholesterol; IL-6: interleukin-6; CRP: C-reactive protein; WBC: leukocytes; NEU: neutrophils; PLT: platelets; FI: fibrinogen; HGB: hemoglobin; sCr: serum creatinine; MA: microalbuminuria; uCr: urinary creatinine; uACR: urinary albumin/creatinine ratio; *p* ≤ 0.05 was considered significant.

**Table 6:** Initial and final testing, intragroup comparison of group D.

	Initial	Final	*p* Value
SBP	152 (143–157.5)	143 (140.5–147.0)	<0.0001
DBP	94.24 ± 2.5	90.16 ± 2.52	<0.0001
ADMA	123 (99–139)	109 (85–114)	<0.0001
HDL	56.96 ± 12.74	70.96 ± 8.33	<0.0001
LDL	110.2 ± 18.80	87.76 ± 10.18	<0.0001
IL-6	2.68 (1.83–4.56)	1.6 (1.15–3.40)	<0.0001
CRP	0.34 (0.17–0.44)	0.2 (0.10–0.30)	<0.0001
WBC	6325 ± 2028	8160 ± 1632	<0.0001
NEU	4770 (3870–5485)	5690 (5230–7230)	<0.0001
PLT	274,080 ± 79,292	250,600 ± 67,775	0.001
FI	3.26 ± 0.75	2.97 ± 0.59	<0.0001
HGB	15.07 ± 1.34	13.66 ± 0.84	<0.0001
sCr	0.84 ± 0.12	0.74 ± 0.13	<0.0001
MA	4.1 (3.2–7.57)	3.3 (2.65–6.4)	<0.0001
uCr	220 ± 63.78	204.2 ± 62.76	<0.0001
uACR	4.08 ± 2.09	2.85 ± 1.53	<0.0001

SD: standard deviation; SBP: systolic blood pressure; DBP: diastolic blood pressure; ADMA: asymmetric dimethylarginine; HDL: high-density lipoprotein cholesterol; LDL: low-density lipoprotein cholesterol; IL-6: interleukin-6; CRP: C-reactive protein; WBC: leukocytes; NEU: neutrophils; PLT: platelets; FI: fibrinogen; HGB: hemoglobin; sCr: serum creatinine; MA: microalbuminuria; uCr: urinary creatinine; uACR: urinary albumin/creatinine ratio; *p* ≤ 0.05 was considered significant.

## Data Availability

The data that support the findings of this study are available upon reasonable request from the corresponding author M.A. The data are not publicly available due to privacy and ethical restrictions.

## Abbreviations

The following abbreviations are used in this manuscript:

SBP	systolic blood pressure
DBP	diastolic blood pressure
IL-6	interleukin-6
ADMA	asymmetric dimethylarginine
AECA	anti-endothelial cell antibodies
HGB	hemoglobin
PLT	platelets
WBC	leukocytes
NEU	neutrophils
HDL	high-density lipoprotein cholesterol
LDL	low-density lipoprotein cholesterol
CRP	C-reactive protein
FI	fibrinogen
sCr	serum creatinine
MA	microalbuminuria
uCr	urinary creatinine
uACR	urinary albumin/creatinine ratio
